# Disruption, control and coping: responses of and to the person with dementia in hospital

**DOI:** 10.1017/S0144686X13000561

**Published:** 2013-09-19

**Authors:** DAVINA POROCK, PHILIP CLISSETT, ROWAN H. HARWOOD, JOHN R. F. GLADMAN

**Affiliations:** * Institute for Person-Centered Care, State University of New York at Buffalo, USA.; † School of Nursing, Midwifery and Physiotherapy, University of Nottingham, UK.; ‡ Health Care for Older People, Nottingham University Hospitals NHS Trust, UK.; § Division of Rehabilitation and Ageing, University of Nottingham, UK.

**Keywords:** dementia, hospital, disruption, control, person-centred care, co-patients, family care-givers

## Abstract

This qualitative study aimed to gain insight into the experience of hospitalisation from the perspectives of the older person with dementia, their family care-giver and other patients sharing the ward (co-patients). Non-participant observation of care on 11 acute hospital wards was supplemented by 39 semi-structured interviews with 35 family care-givers and four co-patients following discharge. Constant comparative analysis produced the core problem facing all those involved: disruption from normal routine meaning that the experience of hospitalisation was disrupted by the presence and behaviour of the person with dementia. Disruption adversely affected the person with dementia, triggering constructive, disengaged, distressed and neutral behaviours. Using Kitwood's model of person-centred care, these behaviours were interpreted as attempts by the person with dementia at gaining a sense of control over the unfamiliar environment and experience. Family care-givers' lives and experiences both inside and outside the hospital were disrupted by the hospitalisation. They too attempted to gain a sense of control over the experience and to give a sense of control to the patient, co-patients and staff. Co-patients experienced disruption from sharing space with the person with dementia and were left feeling vulnerable and sometimes afraid. They too attempted to gain a sense of control over their situation and give some control by helping the person with dementia, the family care-giver and the staff.

## Introduction

In the United Kingdom (UK), people over the age of 65 are the most frequent users of hospital care (Royal College of Psychiatrists 2005; Tadd *et al.* 2011*b*). To put this into context, older people occupy two-thirds of National Health Service (NHS) beds and 60 per cent of those admitted have or will develop a co-morbid mental disorder during hospitalisation. Dementia is the most common psychiatric condition in older people in hospital (31%), followed by depression (29%) and delirium (20%) (Royal College of Psychiatrists 2005). Despite the frequency with which older people are encountered in the system, the quality of hospital care for this group has been poor (Tadd *et al.* 2011*a*). In particular, the care of older people with mental health problems, especially those with chronic (dementia) or acute (delirium) confusion, has been severely criticised in recent years in the media as well as reports by respected organisations such as the Alzheimer's Society UK (2009). As a result, hospitals and their staff have been challenged to improve the situation in response to these criticisms with the support of various policy documents in the UK (Department of Health 2009, 2011; Department of Health, Social Services & Public Safety 2011; National Institute for Health and Clinical Excellence 2006; NHS Confederation 2010; The Scottish Government 2010; Welsh Government 2011). Similar movements in public policy have been initiated internationally (Alzheimer's Disease International 2012).

Research has been published considering the admission of people with dementia to acute settings – from the perspective of staff – as problematic due to issues with the environment (Leung and Todd 2010; Nolan 2007), organisation (Borbasi *et al.* 2006; Eriksson and Saveman 2002) and knowledge of staff (Andersson, Hallberg and Edberg 2003; Pulsford, Hope and Thompson 2007), leading to diminished staff morale (Cocco *et al.* 2003). However, a careful search of the major health science databases revealed only six published studies that focused specifically on the experiences of patients with dementia and/or their family carers in a general hospital setting. One study from Spain, which interviewed family carers one month after their relative had died in hospital, found no difference in the comparative quality of care for patients with dementia and those with heart failure (Formiga *et al.* 2007). However, the remaining five studies all found hospitalisation of the person with dementia to be particularly problematic. A common theme in these studies is the lack of recognition of dementia as a diagnosis and the relationship of this to the quality of care (Cowdell 2010; Norman 2006; Tolson, Smith and Knight 1999). Family carers were distressed by their belief that staff did not understand the condition. Patients with dementia were often noted for their behaviour from ‘unsettled’ to ‘violent’ (Cowdell 2010; Naylor *et al.* 2007; Tolson, Smith and Knight 1999). Poor communication between professionals and agencies and the family carers during hospitalisation led to disputes and upsets between staff and family carers (Douglas-Dunbar and Gardiner 2007; Naylor *et al.* 2007; Norman 2006; Tolson, Smith and Knight 1999) and failed or problematic discharges (Naylor *et al.* 2007; Tolson, Smith and Knight 1999) which caused further upset and dissatisfaction.

The study reported here is part of a programme of research focusing on medical crises in older people, which aims to improve the care of older people in hospital, culminating in a randomised clinical trial of a specialist unit for patients with cognitive impairment admitted to the general hospital for medical care. The study was attached to a cohort study which followed 250 older people admitted to a general hospital who had cognitive impairment (Goldberg *et al.* 2012); and an occupational psychology study of staff caring for the patients with cognitive impairment over a period of six months (Gladman *et al.* 2012*b*).

The significance of this study in comparison with previous studies lies in our attempt to understand the experience of hospitalisation for the older person with dementia in the context of the complex interactions with family carers, other patients and staff. In addition, the experience of patients without cognitive impairment being cared for in the same ward or space, which has not been considered previously, is integrated. This study was designed to develop a theoretical explanation of the experience of the hospitalised elder based on the perspectives of the elder with dementia, their family carer and other patients (co-patients) affected by the hospitalisation.

## Methods

While the involvement of people with dementia in research is a contested area (Bond and Corner 2001), it is argued that, where a ‘safe context’ has been created, such involvement can be beneficial to the person with dementia (Hellstrom *et al.* 2007). However, the philosophical stance underpinning much research conducted within the qualitative paradigm is that the researcher and the researched are equals with equal sharing of information and respect for the participant as the expert in their own experience (Guba and Lincoln 1989). For the researcher exploring the experience of the person with dementia this assumption does not hold so well (McKeown *et al.* 2010). Thus, to design a study that aimed to understand the experience of the person with dementia, the reality that these patients could not always tell us their stories, particularly while acutely ill, or reflect back on their experience in hospital, meant that inevitably there would have to be interpretation: indeed interpretation beyond that normally expected in qualitative research (Riessman 1993). In recognising this position, observation from the tradition of ethnography to watch, interpret and evaluate the experiences of people with dementia on hospital wards was chosen as one of the methods of data collection (Nygard 2006; Tedlock 2000). During these observations the researchers attempted to view the world from the perspective of the cognitively impaired older patient and not just use the perceptions of family members or co-patients or the staff caring for them (Moore and Hollett 2003). In-depth interviews were also conducted with family care-givers (where possible together with the person with dementia), and some co-patients in order to gain different views of the patient's experience as well as the experience of those people most affected by the hospitalisation of the person with dementia. Staff often spoke to the researchers in the course of the observation and field notes from these *ad hoc* conversations are used to support contextual descriptions.

### Sample and recruitment

Recruitment was from two major hospitals that were part of a single NHS Trust within the East Midlands region of the UK. Application to conduct this study was approved by the Research Ethics Committee concurrently with the Better Mental Health cohort study (Goldberg *et al.* 2012). All participants had been recruited to the cohort study and had been admitted to hospital for acute medical care. Typically patients were admitted with a very wide range of medical diagnoses, often associated with a non-specific presentation such as falls, immobility or worsening confusion. All participants for the present study were identified by the researchers on the cohort study, and had some loss of cognitive function using the Mini-Mental State Examination (MMSE). Recruitment occurred over 12 months. Full details are discussed elsewhere (Gladman *et al.* 2012*a*).

### Design

This descriptive exploratory qualitative study used non-participant observation in the hospital setting and semi-structured interviews after discharge at the patients' home to answer the research questions. Observations were undertaken by three researchers: FJ, PC and BR. Interviews were undertaken by two researchers: FJ and PC (see Acknowledgements).

The study involved 72 hours of non-participant observations of care on 45 occasions on 11 wards of the study hospital, including orthopaedic surgery, health care of older people and general medicine. Most observation periods lasted between one and two hours (range 45–180 minutes). Observations were undertaken in various sites on the wards including the multi-bedded bays, patient lounge areas, near the nurses' station and while walking with patients being observed. Most observations ended naturally when the patient was needed for a procedure or the researcher felt the observation had become intrusive. Hand-written field notes were completed during the observations and typed up as soon as possible later (Rodwell 1998).

Observations were complemented by a total of 39 interviews (35 care-givers and four co-patients), concerning the experiences of 38 patients, 29 of whom had dementia and/or delirium, five had other mental health problems and the four co-patients. Interviews were conducted with patients and/or family care-givers at home after discharge. Where the patient had dementia, interview participation was encouraged wherever possible.

The mean age of the patient participants was 86.8 years (range 70–99); 19/34 (56%) were female; 21 (62%) were widowed, nine (26%) were married, two (6%) had never married and two (6%) were divorced. Sixteen had previously lived alone, of whom six returned, eight were discharged to a care home and two died (carers were interviewed). Eleven had previously lived with family, of whom five returned, four went to a care home and two died. Seven had previously lived in a care home, of these three died and the remainder returned to the care home.

The relationship of 32 of the carers to the patient was recorded: wife, nine; daughter, eight; son, seven; niece, two; female friend, two; sister, two; son-in-law, one; and grand-daughter, one. The mean age of carers was 63 (range 46–79) and 24 were female. Of interest, 15 carers disclosed one or more mental health problems of their own.

### Analysis

All interview recordings were transcribed verbatim and anonymised. Pseudonyms were given to all participants. NVivo 8.0 was used (QSR International 2008) for management of data and tracking of analysis. Inter-rater analysis of basic coding was conducted early in the analysis with high levels of agreement (between 68 and 98%). Coding was conducted by DP and PC. Data analysis was based around the principles of the constant comparison method, developed by Glaser and Strauss (1967). Following initial coding of verbatim data, a focused selective phase of analysis involving the synthesis and organisation of the data was undertaken. Finally, more abstract theoretical coding was developed, with hypothesis generation and construction of a substantive theoretical explanation of the experience of hospitalisation for all those involved (Charmaz 2006).

## Findings

The findings aimed to capture the experience of hospitalisation for three groups: (a) the person with dementia (for simplicity we will use patient); (b) their family carer (a role undertaken by a person related by blood, marriage/partnership or friendship); and (c) the co-patients. Data from all three groups and the observations also produced insights into the experience of staff but due to the complexity of that data we have reported it separately (Clissett *et al.* in press).

We present here a description of the core problem, ‘disruption from normal routine’ and the core process, ‘gaining or giving a sense of control to cope with disruption’. The findings reveal that disruption caused by hospitalisation of the patient has the potential to cause loss of personhood for the patient, and an increased risk of vulnerability for the patient, the family care-giver and the co-patient. In order to cope with this disruption, all players try to gain a sense of control over the situation.

### The core problem: disruption from normal routine

The essence of the problem facing the patient is ‘disruption from normal routine’. We found that the concept of disruption from normal routine also could be used to understand the experiences of the family carer and the co-patient. From all these perspectives, when a person with dementia is admitted to hospital there is disruption from what normally happens; behaviours and responses are different and often unanticipated, routines are broken and the consequence of this is difficulty and distress. The problem of disruption does not begin and end with admission to hospital but can be an ongoing series of setbacks, the effects of which can be cumulative. From the interviews we found the beginnings of disruption occurring prior to admission as the patient deteriorated or had an accident that precipitated admission. Disruption continued to occur through the hospitalisation, often lasting beyond discharge, until the patient readjusted to their previous home or new environment.

In the following interview extract, Sally sums up the importance of routine and how her mother's Alzheimer's disease caused more stress during the hospitalisation because of the disruption to her normal routine in the nursing home.

She's now settled, it took her two or three days, but getting her back into the old routine that she had, because with Alzheimer's they've got to stay in a routine, that's the most important thing, that's the only thing they feel comfortable with, is keeping them in a routine, so going to the hospital was out of her routine. Having all these other people bothering her, you know, again the men dressing her, which it wasn't the men's fault, it was there were no females, but yes, the stress was made worse by the Alzheimer's, and that's not the hospital's fault. But don't they have a mental ward there or something? Isn't there anybody there that was equipped to come and deal with it, or come and deal with me so we could both understand a bit better? (Sally, daughter of Victoria)

There was a great deal of disruption for family carers. Not only did they have the worry over the acute illness and the need to get to the hospital each day to visit, but the services which they had in place at home or in the care home were at risk which could cause further disruption, difficulty and vulnerability when the person with dementia was discharged.

Well, I felt on the point of nervous and I can remember feeling as though my head was just going to explode with the worry of it, you know, you've got the stress of seeing your mother dying … I was having to go to the hospital every other day, we took it in turns, my sister went one day and I used to go [the next]. (Brian, son of Hannah)

Another problem was the social services terminated her care package after a fortnight [two weeks] in hospital regardless of what I'd said, and I was keeping in very close contact, keeping them informed, I was very concerned that she should stay with the same carers because she had a relationship with them, they're doing very personal things for her and it worked really well, and I knew she was on, on the brink of not being able to stay at home … (Brenda, daughter of Helen)

The disruption relayed from co-patients was a complicated emotional response because, on the one hand, they recognised the need for the person with dementia to be in hospital but, on the other hand, sometimes found their presence very frightening; leaving them feeling out of control of the situation as described by one co-patient (Anthony) and his wife (Valerie) during their interview at home.

Anthony: As I said, my time resting is part of my healing, I couldn't rest because I was frightened [of the patient with dementia], you know, what's going to happen next…

Valerie: They gave you a buzzer one night didn't they?

Anthony: Oh yes, I had an emergency buzzer put on me just in case; you know – they realised after the first night. The second night they put this emergency buzzer on me so I could call somebody straightaway. I didn't call them for normal things, but only when he began to shake things about, do you know what I mean.

Valerie: When he was pulling the things off the front of the bed wasn't he, and throwing them all over the place.

The following quote from the field notes of an observation serves to indicate disruption to the usual working routine of nursing staff: the time involved in keeping patients safe and the constant interruptions to the work the nurse expects and is expected to do.

The lady in bed 2 stood up. The nurse responded kindly ‘Sit down; I'll come back to you’. The nurse went away and the lady stood up again and started to walk, pushing the table to support her. She walked out of the bay like this. When the nurse noticed her, she approached her ‘Where are you going, where do you want to go? This is not a good idea – you are not really safe. Where do you want to go?’ A colleague gave the lady her walking frame so that she was no longer using the table. Eventually, the nurse said ‘Let's take you back to your bed. We'll take you back to your bed and change your nightie because it's dirty’. At this point, a male patient [also with dementia] who was helping with the menus appeared to attach himself to the lady and the nurse. The lady commented ‘I don't want him with me.’ ‘He's alright, he won't harm you’ said the nurse. The lady tried to push him out of the way and he then tried to hit her in response. The nurse called the man's name and tried to use calm authority to get him to stop. While the nurse was trying to walk with the lady and her walking frame, the male patient appeared to be crowding them out so that the lady kept walking the frame into the wall. Another nurse noticed what was happening and intervened to take the male patient out of the way. She walked up and down the ward with him. While walking, he found another nurse and put his arm around her. She said to him ‘I'd love to walk with you but I have to do the tablets!’ (Field note, ward observation)

Another source of disruption was with the ‘system’ itself. Implicit in family member's comments on the organisation of care was the idea that staff were not adequately trained on how to care for the person with dementia, although by and large the family members interviewed were very sympathetic toward the situation the staff faced.

And maybe, I mean I don't know whether that ward is geared up for dementia patients or whether it's just geared up for old people, certainly some people clearly had got dementia of some description because of the way they were behaving, but some people just looked poorly, you know. So if it's a mixed ward they must have all sorts of jobs on to try to deal with everybody, you know, and maybe the staff don't differentiate between old and frail and dementia, maybe they don't know well enough to know. (Felicity, wife of Edwin)

Some of the worst stories of disruption due to the system were from experiences of admission through the emergency department of being left for hours with no food, nothing to drink and often no pain medications. Family carers were expected to provide care and ensure safety although that often meant they could not go to the toilet or get anything to eat or drink either. For the person with dementia, being treated the same as other patients resulted in a much worse situation:

Well you know like I say, with the medical part once they realised, you know once they saw her and said yes she has broken her hip then it seemed like they started to move. But until that time it was very unnerving, very unsettling, for my mother and for me, by making her wait that long, again it's not necessarily their fault because they were busy, maybe it's their fault that they didn't have enough staff, and there's this [attitude], this is my space [nurses’ station], don't come near it. I so hate that. I asked if I could speak to a doctor – we're busy. And that was in the emergency department … I thought that was totally unacceptable, nobody should be allowed to lay there, especially a person that's mentally impaired, struggling because they don't understand what's happening, they don't understand why they're hurting, and they can't tell you very much because they don't know how to put it into words, and you've got to guess for them, and yet you're made to fight with that person for three or four hours, that's totally stupid. (Sally, daughter of Victoria)

For one nurse who spoke to the researcher during an observation on a ward, the problem was not only the organisation of care but also the philosophy or purpose of the hospital in relation to the person with dementia. The nurse had worked previously in a nursing home.

However, juggling responsibility is a challenge – hospitals are about cure rather than care. ‘Here we cure, in the nursing home we cared’. The nursing team values the ‘softer’ aspects but it tends to take second place to cure. In the nursing home they tended to find that patients were disrupted as a result of being in hospital. There were problems with pressure sores, losing weight and depression when patients returned from hospital. When she worked in the nursing home, she used to phone the wards regularly and be a bit irritating to the members of staff in hospital but now she was beginning to see things from the other side. (Field notes, conversation with registered nurse during observation)

It was clear from the perspectives of family carers, co-patients and staff that the hospital environment was not suited to the patient with dementia. So much so that in both interviews and observations the frustration experienced was palpable. The solutions suggested, as in the following extract from field notes, were recognised as not being ideal as this nursing assistant describes in her conversation with a researcher during an observation:

The other relatives understand but the patients [co-patients] have a low threshold. It's because they're ill. If you feel crap then everything gets your goat – even the phones and the bleeps. They even complain about us talking! So when somebody confused is smearing faeces on your bedcover and putting whoopsies [faeces] in your pillowcase well it creates more than a few harsh words. I mean, I don't like it and I'm paid (laughs). We need an activity room or a special room to nurse the most disruptive, so if they want to piss and poo on the floor then they can, or take their clothes off. I know that's hard and like old times but this is an infection control ward and we can't be always there. It's difficult for their relatives too. (Field notes, conversation with nursing assistant during observation)

The excerpt above highlights the inadequacies of the hospital environment along with some unfortunate attitudes to the patient (implying that patients with dementia choose to behave in a way that is challenging to staff). Since hospitalisation itself may cause disruptive behaviours in the patient with dementia, the lack of both appropriate space and adequately trained staff make it a very difficult environment for all concerned.

### The core process

The core problem gives rise to the core process, which in this study describes the sequence of thoughts and actions revealed by the players in order to cope with the core problem of disruption from normal routine. The core process in this case is to deal with the disruption and is based on a desire for control; that is order, coherence, clarity and calm. In this situation, the assumption is that to feel comforted and secure one has to feel in control of one's life and situation. When a person with dementia is hospitalised, gaining or giving a sense of control is the process that is undertaken by all involved in order to cope with the disruption. In this section the core process is described from the perspective of each group in turn: the patients, family carer and co-patient.

### Actions by the patient to gain or give a sense of control to cope with disruption

The data for this section of the findings come principally from observation of care and represents the greatest level of interpretation as it was not possible ask the patients themselves what was happening or why they were behaving in a particular way. Our understanding was supplemented by interview data and conversations with staff during observations. Our interpretation was that the actions of patients were strategies they used to make sense of their surroundings, for example, asking questions about who people were or where their relatives and belongings were. Activities such as collecting other people's belongings, rearranging drug charts or calling for help can be thought of as making sense of the environment even though those actions might well be seen as disruptive to others. We also noted attempts to take care of family members. However, we found no evidence of actions by patients with dementia that gave a sense of control to either co-patients or members of staff. We interpreted the observed behaviours of patients by taking a person-centred stance (Brooker 2007; Kitwood 1997), meaning that we took the perspective of the person with dementia to interpret the intention or purpose of the behaviour. From this viewpoint we suggest that the patient with dementia gained a sense of control through four categories of behaviour: constructive, disengaged, distressed and neutral. A number of different actions and behaviours were observed within each category. These are summarised in Table 1.

**Table 1.** Actions by the patient with dementia to gain or give a sense of control to cope with disruption

Gaining a sense of control for the themselves	Giving a sense of control to the family carer	Giving a sense of control to the co-patient	Giving a sense of control to the staff
Constructive behaviours: • Sociability • Showing their personhood • Inquisitiveness • Being sociable • Form relationships and attachments • Seeking control • Trying to take control • Being assertive • Resisting • Purposeful activity • Gathering other people's belongings • Work-like activity	Expressions of concern for the family carer, *e.g.* trying to negotiate drinks for the family carer		
Disengaged behaviours: • Exposure • Prolonged inactivity			
Distressed behaviours: • Aggression • Agitation • Challenging the system • Crying • Muttering and moaning • Shouting			
Neutral behaviours: • Causing disruption • Disinhibited use of language • Wandering			

#### Constructive behaviours

Constructive behaviours appeared to be associated with some degree of purpose or positive interaction on the part of the patient with dementia and fit into three broad categories: those involving elements of sociability, those where the person with dementia tried to assert a degree of control over what was happening to them and those where they seemed to be acting in a purposeful manner.

For example, patients appeared to seek companionship even when their communication skills were limited. During a ward observation, Dean and the man in the next bed to him (another patient with dementia) seemed to spend considerable time together, even though it was clear that neither of them could talk in a way that could be easily understood:

A nurse asked Dean if he wanted to get into bed … He appeared to agree and got into bed with assistance. Once he was in bed, the neighbour got up and moved Dean's slippers and sat in the chair right next to Dean's bed. He continued talking to Dean occasionally nudging him. (Field notes, ward observation)

There were a few examples where people seemed to engage in purposeful activity, although this caused disruption to others. Amy reported that her father spent much of his time in hospital gathering other people's belongings but, as she states, this left him in a calm state:

The second time [in hospital] he was calm and collected, and the only trouble was at night time [when] he went round pinching everybody's stuff. He'd got more combs, more toothbrushes and more teeth than anybody else!! He used to go round and fetch everybody's denture fixative, I mean he never used it himself, but he'd got about six tubes of it when he came back, he'd got somebody else's slippers, he's got somebody else's dressing gown. He'd been round all their cupboards and helped himself to their things you see. (Amy, daughter of Ralph)

#### Disengaged behaviours

Disengaged behaviours tended to occur where the patient appeared unaware of the people around and, as a result, engaged in behaviour that either compromised their dignity or wellbeing. We saw this as a form of taking control by withdrawing or being oblivious to those around. One such example was Doris:

She manages to yank the Venflon [intravenous cannula] out with all the tape and bandage attached … Doris then begins dismantling all her bedding and clothing. She is naked within two minutes except for her bed socks, which she pulls up carefully. (Field notes, ward observation)

Another indicator of disengagement seemed to be where the individual had prolonged periods of inactivity, particularly when there were things happening that might have been expected to capture their attention. During an observation, Raymond was sitting at a desk at the end of the bay on the ward:

Raymond sat at this desk for about 30 minutes … at this time there was quite a bit of cheerful chatter between the staff, patients and relatives in this bay but his body language and facial expression indicated that he was not watching or listening to it or that he was in any way interested. (Field notes, ward observation)

#### Distressed behaviours

Distressed behaviours were those that appeared to indicate that the person with dementia was suffering in some way and attempting to make this known. This might simply involve expressions of distress such as crying or rocking or stronger responses such as agitation or aggression.

Jean, a co-patient, commented that she was relieved to be in a side room rather than on the open ward:

I wouldn't have liked to be with them. I would have been more worried about them you know … some of them [were] crying at night. You could hear them crying. (Jean, co-patient)

Bernice experienced a strong reaction of distress from her mother as a result of being in hospital. The sense of control in this case can be interpreted as fighting back in response to the threatening situation in which the patient found herself:

She turned violent, she said ‘I don't want to be here, what they're doing to me is not right, and you shouldn't have brought me in’, and I said, ‘But I didn't bring you in mum’, and she got her stick … and she raised it up and they had to press the button, because she was going to hit me with it. Which she'd never done anything like that in her life. (Bernice, daughter of April)

#### Neutral behaviours

Neutral behaviours were those that were neither positive nor negative in terms of a sense of control for the patient but still may be disruptive to others. They differed from constructive purposeful activity in that it was unclear that the activity was expressing purposefulness on the part of the individual. Typical neutral behaviours included wandering, interfering with other people or their possessions, and disinhibited use of language. One such example was described by Doreen who, when visiting her husband Richard, encountered a woman who appeared to be walking without aim:

There was one woman – I felt sorry for her because she was walking up and down and she said nobody wants me but she was going in the ward she was picking things up, looking. Sometimes she'd put them down or she'd perhaps walk a few steps then she'd come back and put it down again. (Doreen, wife of Richard)

Only a few instances were reported of the patient trying to give a sense of control and comfort to another person and in all cases this was toward the family carer and reported by the family carer in an interview. These actions included expressing concern for the welfare of the family carer and trying to negotiate to promote the comfort of the family carer, for example getting a cup of tea from the tea trolley. Alma stayed a long time in the emergency department with her mother Patricia. She felt that she needed to stay with her because her mother was on a trolley in the middle of the department and Alma was concerned that she might try to get off and fall:

Even when I was standing next to her she'd say, ‘I bet your legs are really hurting you, because I couldn't stand all that time’. And then she'd say to me, ‘Would you like to go and have a drink?’ (Alma, daughter of Patricia)

### Actions by the family carer to gain or give a sense of control to cope with disruption

We found that family carers appeared to take actions to give a sense of control to all players involved in the stay in hospital, including staff. The family carers were instrumental in the core process mainly with regard to the patient and gaining a sense of control for themselves. Family carers were acutely aware of the impact their relatives had on the ward, including the co-patients and staff; they tried to help and shield co-patients and staff from the disruption through their actions. The actions and strategies used by family carers to gain or give a sense of control are outlined in Table 2.

**Table 2.** Actions by the family carer to gain or give a sense of control to cope with disruption

Giving a sense of control to the patient with dementia	Gaining a sense of control for themselves	Giving a sense of control to the co-patient	Giving a sense of control to the staff
Actions to counter system inadequacies: • Advocacy • Filling in major gaps in care • Using detailed knowledge of the person to influence nursing care	Actions to promote coping and change: • Monitoring the quality of care • Complaining and questioning • Coping by becoming expert • Strategising to achieve desired outcomes • Seeking support in order to provide care • Getting things organised while the patient is in hospital	Looking out for the co-patient	Supportive attitude: • Blaming the system not individuals • Expressing support for nurses
Actions to maintain the personhood of the person with dementia: • Showing warmth to the patient • Providing occupation • Promoting dignity for the patient • Maintaining the link with normal life • Substituting for them	Coping by putting things into perspective: • Playing down the seriousness of events • Supporting the NHS and its staff[AQ12] • Blaming the system not individuals • Supporting the nurses • Rationalising behaviour that might be viewed as challenging	Keeping the patient occupied	Supportive actions: • Delivering care to help the nurses • Keeping the patient occupied • Getting involved with co-patients

Family carers attempted to give a sense of control to the patient in two main ways: by acting to counter the inadequacies of the system and by trying to maintain the personhood of the person with dementia.

Three main strategies seemed to be used by family carers to counter the inadequacies of the system: advocacy; using their knowledge of the person to influence care; and getting involved to fill the gaps in care left by the staff and system. Mary found that she was in a position where she had to advocate on behalf of her mother when the hospital system seemed to be a little slow in working towards encouraging the mobility of her mother following a significant fracture:

I was trying to push everybody to get her on her feet, get her back to the care home, given they weren't going to operate or anything. (Mary, daughter of Gillian)

Personhood was preserved by showing warmth at moments of stress, doing things to help keep their relative occupied, respecting the dignity of the person and trying to maintain their link with normal life. Bernice found that the emergency department was quite a challenging environment for her mother. As a result, she did what she could to show warmth and soothe her:

The trolleys really are side by side so you really haven't got much room at all … I stroked her hair and made sure that she was alright. (Bernice, daughter of April)

George: We don't mention dementia in front of him, we just say he forgets and we leave it at that. So because of that I try to get away from him to see the nurse, rather than talk in front of him. You feel sometimes it's like when you had kiddies, but you don't want to feel like that because it's your dad you know.

Janet: We just want to be kind, and not patronise him.

George: It's a bit hard, it's a bit hard really, yes.

Janet: We just try to be respectful to him don't we really.

George: We do indeed, yeah, he deserves that, yes. (George, son of Albert, and his wife, Janet)

We found that family carers had two main strategies to give themselves a sense of control: promoting coping and change by taking actions that helped them feel that they were coping and putting things into perspective which meant choosing to take on attitudes and meanings that allowed them to feel more in control. These strategies included monitoring the quality of care, complaining and questioning, gaining expertise, strategising, seeking support and taking advantage of the time in hospital to get things organised in the community.

One reason for monitoring care quality was because they considered that their relative could not be relied upon to provide an accurate picture of the care that they were receiving. John described the strategy that he used to find out what was happening to his mother Dot:

She doesn't complain much … the trouble really is, because her memory's so poor that … she couldn't tell you anyway if something had happened yesterday or even the same day … So I try and go all sorts of different times of the day … (John, son of Dot)

Another strategy employed by some family carers to gain a sense of control was by rationalising what was happening so that it all seemed to make more sense to them. By doing this, it is possible they were enabled to trust the staff caring for their relative. Putting things in perspective was often required, the playing down of the seriousness of events. For example, when Helen was admitted to hospital, her daughter, Brenda, did not realise that she had had a heart attack – and the hospital staff failed to inform her of this for some time. However, Brenda coped with this by questioning whether or not anything would have been any different had she known this:

They were approachable and I can't really complain other than, I did feel a bit concerned that I hadn't been told that she had in fact had a heart attack. But, as I say, there were no serious consequences of me not knowing. (Brenda, daughter of Helen)

Another way in which some family carers tried to put things in perspective was by rationalising or minimising the behaviour of their relative so that it would seem less troubling or at least they could give an explanation which removed or reduced blame. John reflected on one occasion where his mother became aggressive and reached the conclusion that it was a reasonable response to a situation that she would not have liked:

My mother got a bit confused, and … it's the only time, (laughs) she got a little bit aggressive. I think she just got fed up [because] quite a lot of the time I was having to answer the questions because she wasn't really … So I think being talked across didn't suit her much. (John, son of Dot)

There were a couple of ways that family carers seemed to promote a sense of control for co-patients. These involved looking out for co-patients when they were visiting and keeping their relative occupied during visits. In order to achieve this, our evidence suggests that visitors did not restrict themselves to interaction with their relative only during visiting times. The net result of this seemed to be that visitors would find themselves offering support to patients other than their relative. Alma found that she had to summon the nurse to deal with other patients when she was visiting her mother:

When others were ringing and ringing and ringing for a nurse, there were none coming, you know, or you couldn't find anybody, you went to try and find them yourself. (Alma, daughter of Patricia)

Family carers did try to help the staff cope with their relative and there were two broad strategies that family carers employed that gave a sense of control to members of staff or at least a sense of trust which perhaps helped them to control their anxieties. These were with their attitudes and with their actions. For example, some family carers blamed the government or other agencies for shortcomings in care, removing the spotlight from hospital staff. Brenda targeted her criticism at government targets:

I mean … they're under pressure to get patients out, aren't they? They've got to reach the government's target, and they were making her fit the theory. And I just thought … it's the system isn't it? It's not necessarily the staff. (Brenda, daughter of Helen)

Meanwhile, Diane expressed clear support for nursing staff:

I don't know how they cope. We used to say [this] when we walked out after visiting some nights … some of the nurses would say ‘Well I'm going at 7 o'clock and I'm glad!’ I said I'd be glad for you as well. I felt sorry for them. It's a big responsibility. (Diane, wife of Sidney)

It was clear that some family carers took actions with the specific goal of being supportive to hospital staff. These actions included deliberately choosing to spend more time with their relative so that the person is occupied and not demanding attention from the nursing staff; giving care to the relative along with offering support to co-patients.

Felicity was very clear that her interventions to keep her husband occupied were designed to assist the nursing staff:

I mean the problem with him … was that he wouldn't sit still … he was up and down the ward walking around and I think they [the nursing staff] found this quite troubling. So if I could sit with him and try and get him to stay put that was something for them. (Felicity, wife of Edwin)

### Actions by the co-patient to gain or give control to cope with disruption

Given the level of disruption experienced by co-patients it is not surprising that we found that co-patients needed to take actions to gain a sense of control for themselves. However, there were examples of co-patients also giving a sense of control to others. These are outlined in Table 3.

**Table 3.** Actions by co-patients to gain or give a sense of control to cope with disruption

Giving a sense of control to the patient with dementia	Giving a sense of control to the family carer	Gaining a sense of control for themselves	Giving a sense of control to the staff
• Look out for other patients • Include the person with dementia in visits from their relatives	• Monitor care and report to relatives	• Making the best of things • Being reasonable • Trying to control the behaviour of other patients • Aggression	• Avoid blaming the nurses • Being reasonable

It appeared that some co-patients felt a sense of responsibility for the patients with dementia who were in beds near them, including intervening on occasion. Mike explained that the co-patients in the bay would try to persuade the patient back to their bed area when they looked like they were about to wander off:

You had to try to do something because the nurses didn't have time, so you know people would try to gently guide him to where he should be. (Mike, co-patient)

There was evidence of ambiguity in the relationships between patients and co-patients. There were many expressions of discontent about being placed near someone who was behaving in a way that was perceived to be disruptive. However, there was also evidence of concern for these people on the part of co-patients and even sharing of food at visiting time. During an observation, Bert seemed to be a beneficiary of this:

Bert goes to sit with [co-patient] and helps himself to his biscuits. The man doesn't object and looks blankly as Bert mutters on. A relative arrives and Bert pulls his chair up close. The three of them look as if they are having a close chat even though Bert is not related. The visiting relative seems unconcerned and includes Bert in the general conversation. The biscuits are hoarded into Bert's own pocket. The visitor chuckles and tells his relative he will go down to the shop and get him some more. (Field notes, ward observation)

The main way in which co-patients were able to offer a sense of control to family carers was by being the eyes and ears for the family carer on a 24-hour basis. Many family carers stated that they were unsure of what was happening to their relative on a day-to day-basis because the relative was not able to remember and they did not get clear information from staff. However, there was some evidence that some co-patients would fill them in on a few details. For example, a co-patient informed Mary of moments when the condition of her mother improved:

I mean there were periods later on when she did open her eyes. One of the girls in the bed opposite said ‘Oh, your mum had her eyes open this morning … she was talking’. (Mary, daughter of Gillian)

## Discussion

This qualitative study was embedded in a series of quantitative studies which aimed to contribute to a more comprehensive understanding of the older adult with dementia when hospitalised for physical health problems including the perspectives of family carers and co-patients. The study was limited by the reluctance of co-patients to be interviewed after discharge, leaving us with just four accounts. However, a number of the patients who participated in the study were also co-patients to other people with mental health problems and provided insights into the co-patient perspective. Another limitation was the reliance on observation to reveal the patient's experience rather than interviewing the patient while in hospital. The work of Aggarwal *et al.* (2003), interviewing older adults with dementia, demonstrates this is possible, but in our study the patients' acute illness made it less feasible to add the burden of interview. We chose to interpret actions and behaviours through the lens of Kitwood's (1997) philosophy of person-centred care as a way of substantiating our interpretation. Kitwood emphasises the need to see the world from the perspective of the person with dementia and that is what we have tried to do.

Our analysis of the data revealed a core problem and a core process. The core problem was that the ‘disruption from normal routine’ caused by illness and admission was a major source of stress for the patient. In order to discuss what we mean by ‘disruption from normal routine’ we must consider first the importance of routine to the person with dementia and the family care-giver. Familiarity and routine are key elements of daily care for the person with dementia. Maintaining a familiar environment and avoiding or planning carefully for changes in routine are simple but effective strategies recommended by care-giver advice and support groups and dementia-related charities (*see e.g.* Mayo Clinic 2012). In their review examining research solely focused on presenting the perspective of the person with dementia, de Boer *et al.* (2007) found that the principal ways in which people with dementia tried to continue to live as well as possible were to stick to daily routines and to maintain meaningful relationships. By doing this they maintain control over their lives.

Dementia does not overwhelm the person suddenly. It has an insidious beginning and a progressive course, which can allow the person to adapt gradually to their changing situation (de Boer *et al.* 2007). Thus the preference for routine is understandable, as is the need for carers to plan for change. Hospitalisation represents swift and unmitigated disruption to routines and the possibility of slow adaptation is negated. Hospitalisation is sudden and necessitates several distinct changes in surroundings (home, ambulance, emergency room, medical admissions unit and finally the ward) as well as distinctly different and unfamiliar people doing unfamiliar and frightening things. In addition, the person with dementia is probably not feeling well, being acutely ill, perhaps in pain or experiencing other physical symptoms and even delirium.

In the unfamiliar environment of the hospital, the patient with dementia tries to make sense of his/her world, a process we called ‘gaining or giving a sense of control to cope with disruption’. The core process represents the basic social psychological process at play in the experience (Charmaz 2006). We found that the three groups (patient, family carer and co-patient) were all attempting to gain a sense of control for themselves or give a sense of control to members of the other groups including the staff. The purpose of gaining/giving a sense of control is to minimise the impact of the disruption caused by hospitalisation. Feeling in control is clearly an outcome of coping under difficult and unusual circumstances. Participants displayed both emotion-focused and problem-focused strategies very much in tune with those proposed by Lazarus and Folkman (1984). These overall findings clearly echo work by Swedburg *et al.* (2012), who also found that highly dependent older people living in the community felt insecure and tried to gain a sense of safety, security and control when learning to live with the disruption of nursing assistants coming to the house to provide care.

However, it should be noted that this is a dynamic and interactive process. The data suggest that the admission of someone with dementia into hospital is disruptive to all parties (patients, co-patients, family carers and staff). None of the parties exist in a vacuum – the way in which one actor seeks to gain a sense of control has an impact on the extent of disruption experienced by another, possibly the way in which they might seek to gain a sense of control and, probably, the outcomes.

**Figure 1.**
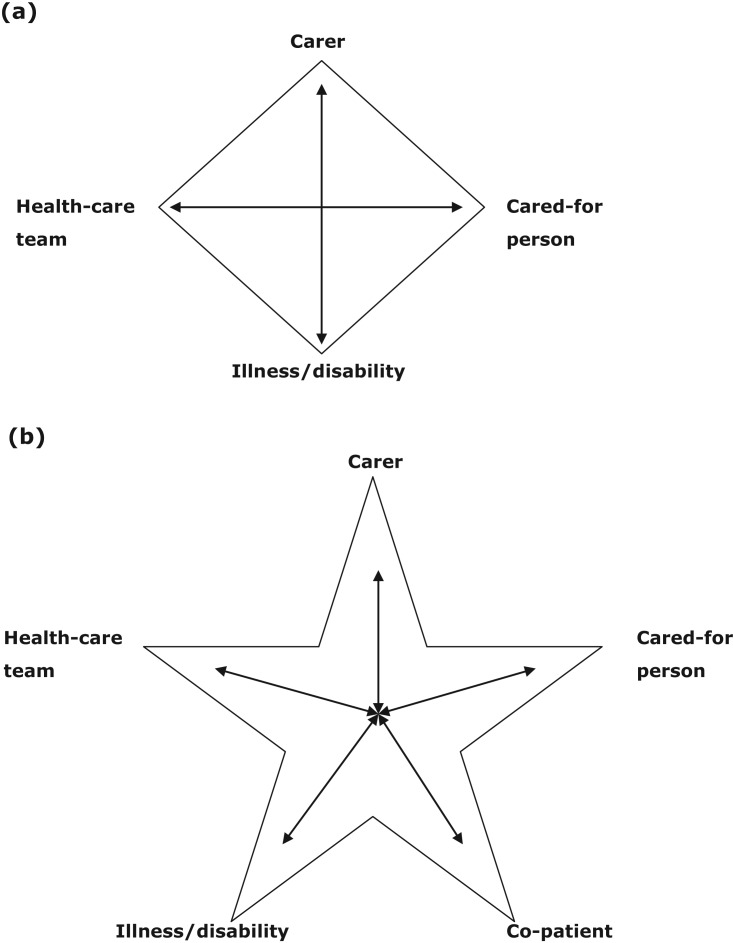
(a) The therapeutic quadrangle. (b) The therapeutic star. *Source*: (a) Rolland (1994: 57).

These findings can be better understood when considered in the light of two theoretical concepts: the therapeutic quadrangle (Rolland 1994) and Systems Theory (Dallos and Draper 2000). The therapeutic quadrangle is illustrated in Figure 1a.

Rolland (1994) argues that to give the subject of care-giving effective consideration, all four elements of the quadrangle must be considered: the person receiving the care, the family carer, professional care-givers and the nature of the illness. The interaction of these four elements influences the experience of giving or receiving care. However, we suggest that, in in-patient settings, there is a fifth element to this ‘quadrangle’: the co-patient. The findings indicate that the relationship between the co-patient and the patient affects the way in which all parties experience the admission to hospital of the person with dementia. As argued in the remaining sections, the added complexity of the co-patient interactions causes a change in the experience of care, therefore the illustration proposed is modified for the in-patient setting to a five-pointed star (Figure 1b). This acknowledges the interactions with co-patients, which complicates care both for the recipients of care and for professional and family care-givers.

It is also useful to consider these findings within the framework of systems theory. Bateson (1972) defined a system as being any unit that is structured on feedback. It consists of a set of interacting parts which communicate on a mutual basis resulting in each part influencing and being influenced by the other, ultimately displaying identifiable coherent patterns (Dallos and Draper 2000). These coherent patterns provide a stable context for individual and mutual functioning (Jones 1993) – *i.e.* although systems may be influenced by external forces, there is a tendency for the system to reach a state of dynamic equilibrium (Wadsworth 2008).

Data from this study also suggest that, for many participants, the disruption of admission to hospital is an external force that has a strong negative influence on the functioning of the family system. Admission into hospital puts many people with dementia into a situation where they spend 24 hours a day interacting with a number of co-patients. It is reasonable to consider this to be a new ‘system’ for the period of time in hospital. However, a number of factors might either inhibit the extent to which this new system can reach stability, or contribute to making this system a negative experience for both patient and co-patient. Firstly, there is the response of the person with dementia to a disruption – which results in disengaged or distressed behaviour – which in turn induces a response from the co-patient. Secondly, the co-patient who is coping with their own illness and hospitalisation is less able to respond constructively to any challenges presented by the behaviour of the person with dementia and reacts accordingly. Thirdly, the nature of the illness of both patient and co-patient may inhibit the evolution of the relationship, especially if there is unpredictable behaviour or disease progression with either party.

**Figure 2.**
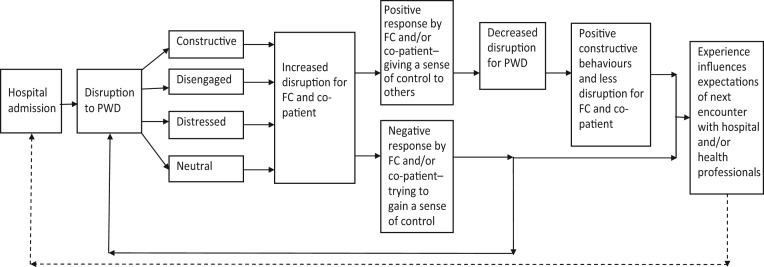
Flow chart of the hypothesised impact of person with dementia (PWD) disruption on the family carer (FC) and co-patients.

The patients with dementia appeared to seek a feeling of control by constructive, disengaged, distressed or neutral behaviours. Each behaviour type has an impact on the disruption experienced by family carers and co-patients. For co-patients, this included feeling a sense of responsibility for the patients with dementia who were in nearby beds, which resulted in their intervention on occasions and being the eyes and ears for the family carer on a 24-hour basis. There were other less positive responses by co-patients that had the potential to increase the sense of disruption experienced by the person with dementia. This is illustrated in Figure 2.

We argue that family carers are likely to have very little influence on the nature of the interaction between the patient with dementia and co-patient. However, this interaction will have an impact on the extent of disruption experienced by the family carer and their responses to this disruption, most notably on whether they focus their energies on gaining a sense of control for themselves, the person with dementia, the co-patient or member of staff. Here we propose that these triadic interactions can be categorised into four different types: harmonious, rubbing along, disruptive and dysfunctional. Table 4 details the type of interaction, the impact on the relationships between the person with dementia, the family care-giver and the co-patient, and in the final column our proposed impact on the care provided by health professionals in response.

**Table 4.** Types of interactions between the person with dementia, the family carer and the co-patient

Type of triadic interaction	Impact on person with dementia	Impact on health-care professionals
All working together	PWD displays positive constructive behaviours, and FC and co-patient act to give each other a sense of control	Can focus on giving good dementia care
Rubbing along	PWD displays constructive behaviours which challenge disengaged, distressed or neutral behaviours but FC and co-patient continue to act to give all parties a sense of control	Can focus on giving good dementia care but this is more challenging
Disruptive	PWD displays constructive behaviours which challenge disengaged, distressed or neutral behaviours and the co-patient responds aggressively but FC attempts to act to give all parties a sense of control	Need to consider issues of safeguarding and increased disruption for PWD and protection of co-patient
Dysfunctional	PWD displays constructive behaviours which challenge disengaged, distressed or neutral behaviours and the co-patient responds aggressively and FC seeks to gain a sense of control	Facing a multiplicity of demands

*Notes*: FC: family carer. PWD: person with dementia.

The interventions of health-care professionals take place in the context of these complex sets of relationships. Where the triadic relationship is dysfunctional, the challenges faced by health-care professionals are significantly greater than situations where the relationship is harmonious.

It has been accepted that health-care professionals who care for people with dementia in acute care settings need the skills to work with people with dementia as well as the clinical skills to address the primary cause of admission to hospital (Clissett *et al.* in press; Moyle *et al.* 2008). We argue that, in addition to this, health-care professionals need to have insight into the complexities of the interactions and relationships between the patient, the co-patient and the family care-giver; an ability to ‘read’ these interactions in any particular situation; and an understanding of the interventions that will help all parties gain a greater sense of control and reduced feelings of disruption.

## Conclusion

This study adds to our knowledge of the care experience for the person with dementia when they are admitted to hospital and does so, not in isolation from other parties, but by illustrating the complex interactions that occur. Two main messages come from our data. First of all the recognition that disruption from normal routine does not just affect the person with dementia in hospital but also affects the way in which family care-givers interact with their relative, the staff and the other patients in the ward. Similarly, the staff's work is disrupted and their interactions with the patient are not routine which affects the relationship and interactions with the patient's family care-givers and in turn alters the interactions and attention given to co-patients. The second message is that without adequate understanding through training or without adequate change in the system, hospital health professionals will continue to be left at a disadvantage in caring for this most vulnerable population.
